# In Silico, In Vitro, and Clinical Investigations of Cathepsin B and Stefin A mRNA Expression and a Correlation Analysis in Kidney Cancer

**DOI:** 10.3390/cells11091455

**Published:** 2022-04-25

**Authors:** Magdalena Rudzinska-Radecka, Anastasia S. Frolova, Anastasia V. Balakireva, Neonila V. Gorokhovets, Vadim S. Pokrovsky, Darina V. Sokolova, Dmitry O. Korolev, Natalia V. Potoldykova, Andrey Z. Vinarov, Alessandro Parodi, Andrey A. Zamyatnin

**Affiliations:** 1 Institute of Molecular Medicine, Sechenov First Moscow State Medical University, 119991 Moscow, Russia; magdda.rudzinska@gmail.com (M.R.-R.); frolanasta@gmail.com (A.S.F.); balakireva.anastacia@gmail.com (A.V.B.); gorokhovets@gmail.com (N.V.G.); aparodi.sechenovuniversity@gmail.com (A.P.); 2 Institute of Physical Chemistry, Polish Academy of Sciences, 01-224 Warsaw, Poland; 3 Department of Biotechnology, Sirius University of Science and Technology, 1 Olympic Ave., 354340 Sochi, Russia; vadimpokrovsky@gmail.com (V.S.P.); d.v.sokolova@gmail.com (D.V.S.); 4 Shemyakin-Ovchinnikov Institute of Bioorganic Chemistry, 117997 Moscow, Russia; 5 Laboratory of Combined Treatment, N.N. Blokhin Cancer Research Center, 115478 Moscow, Russia; 6 Department of Biochemistry, Peoples’ Friendship University of Russia (RUDN University), 117198 Moscow, Russia; 7 Institute for Urology and Reproductive Health, Sechenov University, 119992 Moscow, Russia; demix84@inbox.ru (D.O.K.); potoldykovanv@gmail.com (N.V.P.); avinarov@mail.ru (A.Z.V.); 8 Belozersky Institute of Physico-Chemical Biology, Lomonosov Moscow State University, 119992 Moscow, Russia; 9 Department of Immunology, Faculty of Health and Medical Sciences, University of Surrey, Guildford GU2 7XH, UK

**Keywords:** cathepsin B, stefin A, renal cell carcinoma, human specimens

## Abstract

The cysteine protease Cathepsin B (CtsB) plays a critical role in multiple signaling pathways, intracellular protein degradation, and processing. Endogenous inhibitors regulate its enzymatic activity, including stefins and other cystatins. Recent data proved that CtsB is implicated in tumor extracellular matrix remodeling, cell invasion, and metastasis: a misbalance between cathepsins and their natural inhibitors is often considered a sign of disease progression. In the present study, we investigated CtsB and stefin A (StfA) expression in renal cell carcinoma (RCC). mRNA analysis unveiled a significant *CTSB* and *STFA* increase in RCC tissues compared to adjacent non-cancerogenic tissues and a higher CtsB expression in malignant tumors than in benign renal neoplasms. Further analysis highlighted a positive correlation between CtsB and StfA expression as a function of patient sex, age, tumor size, grade, lymph node invasion, metastasis occurrence, and survival. Alternative overexpression and silencing of CtsB and StfA confirmed the correlation expression between these proteins in human RCC-derived cells through protein analysis and fluorescent microscopy. Finally, the ectopic expression of CtsB and StfA increased RCC cell proliferation. Our data strongly indicated that CtsB and StfA expression play an important role in RCC development by mutually stimulating their expression in RCC progression.

## 1. Introduction

Renal cell carcinoma (RCC) is one of the most common urogenital tumors [1], and according to histological characterization, is classified as clear cell (KIRC; accounting for 75–80% of all RCC cases), papillary (KIRP; 10–15%), and chromophobe (KICH; 5%) renal cell carcinoma [2]. Despite recent progress in early RCC detection and treatment, the patients diagnosed with advanced disease are still affected by a high mortality rate [3]. Moreover, 20–30% of the cases present metastases [4]. In this scenario, more investigations on RCC pathogenesis and progression, novel pharmacological targets, and valuable diagnostic markers are needed [5].

Endosomal proteases might play a crucial role in tumor metabolism [6], proliferation [7], and distant organ invasion [8,9,10]. Cysteine cathepsins (Cts) are cellular proteases generally located in the lysosomes [11] even though they have also been detected in other cellular compartments, including the cytoplasm [12], membrane [13], and nucleus [14]. CtsB has been extensively investigated in cancer disease [15]. After translation, the immature protein undergoes different proteolytic steps [16] to direct the protein to the lysosomal compartment and generate the mature enzyme consisting of a heavy (25 kDa) and a light chain (5 kDa) [16,17] (Supplementary Figure S1A).

The Cts activity is controlled by endogenous peptidase inhibitors that interact reversibly or irreversibly with their active site. Among them, the most essential is the cystatin superfamily, including evolutionarily related proteins expressed in all living organisms [18]. Type I cystatins (stefins) are not glycosylated proteins detected intracellularly and in body fluids. A high concentration of Stefin A (StfA) was found in polymorphonuclear leukocytes [19] and epithelial cells [20] in which StfA was involved in the prevention of apoptosis and epidermal development/maintenance, respectively. This competitive inhibitor reversibly suppresses CtsB proteolytic activity (inhibition constant vs. CtsB = 10) [21] (Supplementary Figure S1B) via weak interactions followed by a conformational change of the enzyme [22].

CtsB overexpression has been detected in many types of malignant tumors (e.g., prostate [23], pancreatic [24] cancers, and melanoma [25]), including RCC [26], indicating its potential value as a therapeutic target. On the other hand, reduced CtsB expression has been shown to reduce glioma [27], osteosarcoma [28], and mammary cancer [29] cell malignancy, while a low StfA expression was associated with glioblastoma [30], breast [31], and head and neck cancer progression [32]. Contrasting evidence has also been reported despite a large consensus correlating a higher malignant behavior with increased proteolytic activity. In a murine squamous cell carcinoma model, CtsB knockdown failed to affect tumor progression [33] while increased StfA expression in breast [34], liver [35], and brain [36] cancer was associated with decreased survival.

While the disbalance between CtsB and StfA may represent a reliable disease progression marker, this phenomenon should be explored in the specific context of tissue and cancer type, and to our knowledge, no previous studies have investigated CtsB and StfA in RCC. We hypothesize that CtsB and the expression of its endogenous inhibitor StfA could change significantly in diseased tissue and could correlate with clinical data. In this work, we studied the mRNA expression pattern of CtsB and StfA in a collection of RCC tissues and paired non-tumoral specimens. This analysis registered an overall increased *CTSB* and *STFA* mRNA expression but not an increase in the *CTSB*/*STFA* ratio. In addition, cancer mRNA expression correlated with some patient clinicopathological characteristics. The same analysis performed on a small group of patient samples and in vitro on human RCC-derived cell lines indicated that the expression of these two proteins is reciprocally dependent and can affect RCC cell proliferation. Our observations generated an additional understanding in validating CtsB, StfA, or their ratio as tumor progression biomarkers and their impact on RCC malignancy.

## 2. Materials and Methods

### 2.1. Renal Cancer Tissue Sampling and Cell Lines

The tissues obtained for this study included RCC (T) specimens and the surrounding non-cancerous (NT) tissues. The samples were collected from patients with renal angiomyolipoma (AML; *n* = 3 (7%)), pT1-2N0M0 renal masses treated with laparoscopic and robotic partial nephrectomy (*n* = 26 (60%)), and patients with pT3-4 N0-1 M0-1 renal masses treated with laparoscopic radical nephrectomy (*n* = 14 (33%)) at the Institute for Urology and Reproductive Health, Sechenov University, Moscow, Russia between 2019 and 2020. The research was performed following the ethical guidelines approved by the Ethical Committees of Sechenov University (Moscow, Russian Federation). Tissues were collected from 43 patients (22 males and 21 females), with a median age of 58.8 years (age range between 26 and 80 years). Six patients had developed distant metastasis (including metastasis to the lung, liver, bones, and adrenal gland), and two presented paraaortic lymph node involvement. The postoperative histopathological verifications of RCC samples were performed according to the International Agency for Research on Cancer (2016) WHO classification of tumors of the urinary system and male genital organs (IARC WHO classification of tumors), 4th ed. Written informed consent was received from all the patients before their inclusion in the study. The study was conducted following the Declaration of Helsinki, and the Ethics Committee approved the protocol at Sechenov University, Moscow, Russia, N04-12.

The cell lines derived from human renal cancer, 769-Pand A498 were purchased from American Type Culture Collection. The cells were grown in RPMI 1640, supplemented with 10% fetal bovine serum and a 1% mixture of penicillin–streptomycin antibiotics (all from Gibco, Waltham, MA, USA) at 5% CO_2_ and 37 °C in a humidified atmosphere containing 5% CO_2_. Cell lines were authenticated by STR DNA Profiling Analysis (GORDIZ, Moscow, Russia). Cell lines were checked with the MycoAlertTM Mycoplasma Detection Kit (Lonza, Basel, Switzerland) and were free of contamination.

### 2.2. RNA Isolation and Real-Time Polymerase Chain Reaction (RT-qPCR)

Total RNA was extracted from kidney specimens (tumoral and adjacent healthy tissue) using the Total RNA isolation kit (Evrogen, Moscow, Russia), following the manufacturer’s protocol. Next, complementary DNA (cDNA) was transcribed from mRNA using a cDNA synthesis kit (Evrogen, Moscow, Russia). For the reverse transcription reaction, one µg of total RNA was used with optical density OD260/OD280 1.7–2.0 measured with NanoDrop One (ThermoFisher, Waltman, MA, USA). Expression of the human genes was quantified by RT-qPCR using the cDNAs as templates in reactions containing the double-stranded DNA-specific dye BioMaster HS-qPCR SYBR Blue (2×) (BiolabMix, Novosibirsk, Russia) and specific oligonucleotide primers (*CTSB*: F-5′-TTCTTGCGACTCTTGGGACTTC-3′, R-5′-TGACGAGGATGACAGGGAACTA-3′; *CTSB*: F- 5′-AAACCCGCCACTCCAGAAAT-3′, R-5′-TTTGTCCGGGAAGACTTTTG-3′; *GAPDH*: F-5′-CTTCGCTCTCTGCTCCTCCTGTTCG-3′, R-5′-ACCAGGCGCCCAATACGACCAAAT-3′). PCR reactions were performed in triplicate with the following conditions: 95 °C/30 s, 40 cycles of 95 °C/5 s, 60 °C/15 s, and 72 °C/10 s in the iQ5 Real-Time PCR Detection System (Bio-Rad, Hercules, CA, USA). The quantification cycle (Cq) values estimated for analyzed genes were normalized against the corresponding Cq values of *GAPDH*. The relative quantification value (RQ) was calculated as the relative change in *CTSB* or *STFA* transcript expression level compared to the level of these genes in the internal control represented by a mixture of RNA extracted from control samples.

### 2.3. Transfection

#### 2.3.1. *CTSB* and *STFA* Overexpression in RCC-Derived Cells

769-P and A498 cells were grown up to 70% confluence, washed with Dulbecco’s phosphate-buffered saline (D-PBS), trypsinized, centrifuged, and then re-suspended in a fresh medium with 10% FBS. Cells were transiently transfected with pcDNA-3.1-*CTSB* or pcDNA3.1-*STFA* plasmid; an empty plasmid served as a control (pcDNA-3.1; ThermoFisher, Waltham, MA, USA). Human *CTSB* cDNA was subcloned into a eukaryotic expression vector pcDNA3.1-*CTSB* in the sense orientation, and a plasmid with *STFA* sequence (pcDNA3.1-*STFA*) was a kind gift from Dr. Yuan Chen, Universitätsklinikum J07747 Jena, Germany.

Cells were transfected with pcDNA 3.1-*CTSB*, pcDNA3.1-*STFA* (also called pl*CTSB* and pl*STFA*), or empty plasmid (referred to as CTRL) using Lipofectamine 3000 (Invitrogen, Carlsbad, CA, USA) according to the manufacturer’s instructions on 6-well, 24-well, or 96-well plates. Cells were harvested at 48, 72, and 96 h following transfection. The transfection efficiency was verified using Western blotting. The highest CtsB and StfA expression was observed after 72 h. The experiment was repeated at least three times with similar results.

#### 2.3.2. Silencing of *CTSB* and *STFA* in RCC-Derived Cells

Cells were grown to 60–70% confluence and transiently transfected with short hairpin RNAs (shRNAs) to induce the silencing of *CTSB* or *STFA*. shRNA sequences used for silencing included: human *CTSB*: F-5′-TGAATTCCCAACACGTCACCGGAGAGATAAGATCTAAT-3′, R-5′-ATAGTCGACCCAACACGTCACCGGAGAGATTAGATCTTAT-3′, human *STFA*: F-5′-TGAATTCAGGTACGAGCAGGTGATAATAAGATCTTATT-3′, R-5′-ATAGTCGACAGGTACGAGCAGGTGATAATAAGATCTTATT-3′. These sequences were cloned into the pCI-neo (Promega, Madison, WI, USA) using EcoR1 and SalI. According to the manufacturer, plasmids were used to transfect 769-P and A-498 cell lines for 48, 72, and 96 h using Lipofectamine TM 3000 (Invitrogen, Carlsbad, CA, USA)’s instructions. The knockdown efficiency was verified using Western blotting, and the strongest reduction in analyzed proteins was observed after 72 h of transfection; the experiment was repeated at least three times with similar results.

### 2.4. Western Blotting

The cells after the transfections were washed twice with ice-cold PBS, harvested, and re-suspended in a lysis buffer 50 mM Tri-HCl (pH 8.0), 100 mM NaCl, 0.5% NP-40, 1% Triton X-100, 1× protease inhibitor cocktail (ThermoFisher, Waltham, MA, USA), supplemented with 1% protease inhibitor cocktail (Roche, Basel, Switzerland). Proteins in the cell lysates were quantified, and samples of 30 µg were resolved on 12% SDS-PAGE gels and then transferred to the PVDF membranes (Bio-Rad, Hercules, CA, USA). The expression of CtsB and StfA was detected using specific primary antibodies (ab190077 and ab188502 Abcam, Cambridge, UK). After intensive washing, the membranes were incubated with (P-GAR Iss (Goat pAb to rabbit IgG (HRP), Abcam, UK; 1:5000) in 5% non-fat milk in PBST. Signals from reactive bands were visualized by enhanced chemiluminescence detection (Bio-Rad, USA). As a loading control, the membranes were incubated with a primary anti-tubulin antibody (1:5000; Ab52866, Abcam, UK) and secondary Rabbit Ab to mouse (Abcam, UK; 1:5000), identically.

### 2.5. Immunofluorescence Staining

The cells 72 h after transfection were washed with D-PBS, fixed in 4% PFA/PBS for 15 min, and permeabilized in 0.25% Triton^®^ X-100 for 10 min. After blocking the non-specific sites in 2% BSA/PBS-T, the immunofluorescence was performed overnight with primary antibodies described in the Western blotting section. Next, cells were incubated for 1 h, RT with the fluorophore-labeled secondary antibody Donkey anti-Rabbit IgG (H + L) ReadyProbes™ (ThermoFisher, USA). The cells were then counterstained with 0.5 µg/mL of nuclear dye DAPI (Sigma-Aldrich, Saint Louis, MI, USA) and visualized under a confocal microscope (AxioObserver Z1, Zeiss, Oberkochen, Germany) using oil-immersion lenses.

### 2.6. Cell Proliferation Assay

RCC-derived cell lines were plated in 96-well plates overnight, and the cells were transfected with constructs using Lipofectamine 3000 reagent. At 48 h post-transfection, cells were further cultured in RPMI-1640 supplemented with 10% FBS for 72 h before adding MTT to each well. Following incubation with (3-(4,5-dimethylthiazol-2-yl)-2,5-diphenyltetrazolium bromide) (MTT) for six h at 37 °C, the MTT solution was removed and exchanged for MTT solvent (4 mM HCl, 0.1% NP40 in isopropanol) and placed on an orbital shaker for 15 min. Each well’s optical density (OD) was measured at a wavelength of 490 nm using an ELISA microplate reader (BMG Labtech, Ortenberg, Germany). The experiments were performed in five replicates and repeated at least three times.

### 2.7. Statistical Analysis

Statistical analysis was performed using GraphPad, Prism 6.00 for Windows, Graf Pad software San Diego, CA, USA. To compare independent groups, ANOVA Kruskal–Wallis, Student’s *t*-test, or U Mann–Whitney test were used. To evaluate the relationship between *CTSB* and *STFA* expression levels and patients’ characteristics (age and sex of the patient) and the clinical features of the tumor (staging according to tumor node metastasis (TNM), American Joint Committee on Cancer Staging (AJCC) and histopathological RCC subtypes) the Spearman’s rank correlation was applied. The results of relative expression analysis are presented as mean ± SD for normal distribution. The *p*-value < 0.05 was considered statistically significant with * *p* < 0.05, ** *p* < 0.01, and *** *p* < 0.001.

### 2.8. Database Analysis

The online database Gene Expression Profiling Interactive Analysis (GEPIA; http://gepia.cancer-pku.cn/index.html, accessed on 1 January 2022; [37]) is a communal network server that includes a transcriptome sequencing dataset of 9736 tumors together with 8587 adjacent normal tissues from The Cancer Genome Atlas (TCGA) and Genotype-Tissue Expression (GTEx) data sets. Here, the genes’ signature correlation was validated in the GEPIA platform using the Pearson correlation coefficient method (KICH, *n* = 66; KIRC, *n* = 523; KIRP, *n* = 286 and NT, *n* = 100). Using the TCGA (The Cancer Genome Atlas/Pathology Atlas) database (https://tcga-data.nci.nih.gov/tcga/, accessed on 1 January 2022; [38]), the *CTSB* and *STFA* expression was correlated with the survival of RCC patients. The Kaplan–Meier plots log-rank allowed the predictive value of the analyzed genes to be analyzed.

## 3. Results

### 3.1. Overall CtsB and StfA mRNA Expression Increased in RCC Specimens and Was Associated with Some Clinicopathological Characteristics

Forty-three RCC patients were enrolled in the study, and a *CTSB* and *STFA* transcript analysis was performed on primary RCC (T) tissues and surrounding non-cancerous kidney (NT) (Supplementary Table S1).

**Figure 1:**
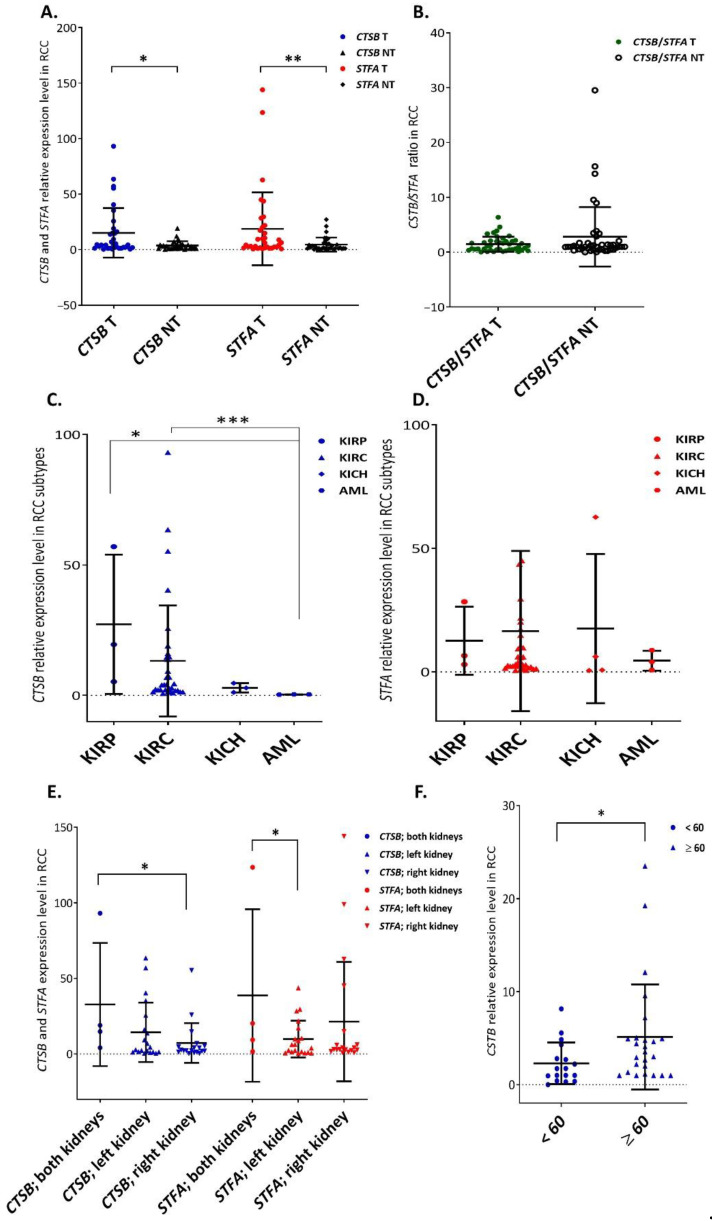
*CTSB* and *STFA* mRNA expression in human renal cell carcinoma and surrounding non-tumoral tissue. (**A**) *CTSB* and *STFA* were significantly (Student’s test, *CTSB*, *p* = 0.012; *STFA*, U Mann–Whitney test, *p* = 0.007) increased in tumor tissues; (**B**) *CTSB*/*STFA* ratio was lower in tumors than in healthy kidneys (*p* > 0.05); CTSB/STFA represents CTSB expression/STFA expression in the same tissue, tumor (T) or non-tumor sample (NT); (**C**) *CTSB* mRNA expression was significantly higher in papillary cell renal (KIRP) and clear cell renal cell (KIRC) carcinomas compared to renal angiomyolipoma (AML; Kruskal–Wallis test, *p* = 0.03, *p* = 0.009, respectively); (**D**) *STFA* did not increase significantly in KIRP, KIRC, and KICH compared to AML (*p* > 0.05); (**E**) in both kidneys *CTSB* and *STFA* were significantly (UMann-Whitney test, *CTSB*, *p* = 0.027 and *STFA*, *p* = 0.036) up-regulated compared to tumors occurring only in right or left kidney, respectively; (**F**) *CTSB* mRNA expression increased in surrounding non-cancerous kidney tissues of elders (U Mann–Whitney test, *p* = 0.0268). Results represent the mean ± SD. * = *p* < 0.05, ** = *p* < 0.01, *** = *p* < 0.001.

Within all the considered parameters, RT-qPCR showed a significantly higher expression of both genes (*CTSB*, *p* = 0.012; *STFA*, *p* = 0.007) in cancer tissues (T) than in surrounding healthy kidneys (NT) (Figure 1A). Compared to CtsB, the increase in *STFA* expression in cancer tissues was higher than in control specimens, decreasing the *CTSB*/*STFA* ratio in the tumor (Figure 1B).

*CTSB* expression significantly increased in KIRC and KIRP cancers compared to the benign renal neoplasm—renal angiomyolipoma (AML, *p* = 0.03; *p* = 0.009, respectively), while KICH did not show any remarkable difference (Figure 1C). On the other hand, *STFA* increased in all the malignant phenotypes compared to AML, without reaching statistical significance (Figure 1D). Next, when cancer lesions occurred in both the kidneys, *CTSB* and *STFA* expression significantly increased compared to tumors occurring in the right (*CTSB*, *p* = 0.027; Figure 1E) or the left kidney (*STFA*, *p* = 0.036; Figure 1E), respectively. Finally, in non-tumor tissues, the *CTSB* expression was remarkably (*p* = 0.0268) enhanced in older patients (>60 years vs. ≤60 years; Figure 1F).

Compared to non-tumoral tissue, the changes in *CTSB* and *STFA* expression correlated, both increasing and (in some cases) decreasing, as shown by Spearman’s rank correlation analysis (Table 1 and Supplementary Figure S2). A positive correlation between *CTSB* and *STFA* expression was also detected in tumors isolated from patients without metastases. Interestingly, a negative correlation between the expression of these proteins was detected comparing tumor *CTSB* expression and healthy tissue *STFA* expression: a higher *CTSB* mRNA content in tumors was accompanied by a lower *STFA* mRNA level in paired non-tumor samples.

Furthermore, according to the Pathology Atlas database, the up-regulation of *CTSB* and *STFA* correlated with the shorter survival of KICH, KIRP, and KIRC patients (Supplement Figure S2).

### 3.2. Cathepsin B and Stefin A mRNA and Protein Expressions Showed a Positive Correlation Pattern in Renal Cell Carcinoma and Non-Tumoral Kidney Tissues

**Figure 2:**
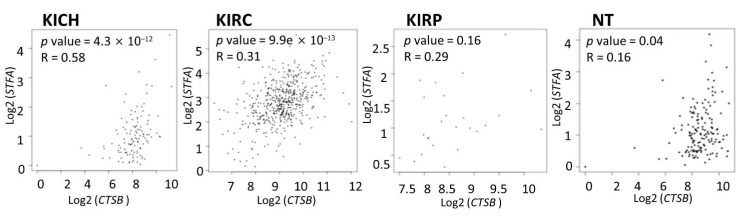
*CTSB* and *STFA* share similar expression patterns in renal cell carcinoma and normal kidney tissues (NT) ranked by the Pearson correlation coefficient. GEPIA provides a pairwise *CTSB*/*STFA* gene correlation analysis for given sets of TCGA and/or GTEx expression data in the correlation graphs. Individual points upon which the correlation is calculated are a single value of multiple samples.

To corroborate the evidence of the positive *CTSB*/*STFA* correlation, we analyzed the expression of these genes in a bigger cohort of RCC patients using the GEPIA online database. As a result, *CTSB* and *STFA* showed a positive correlation in all subtypes of RCC and normal kidney tissues (Figure 2). Correlation analysis was performed on the selected genes in a pairwise manner. The Pearson coefficients were automatically calculated highlighting a significant *CTSB* and *STFA* correlation in KICH (*p* = 4.3 × 10^−12^), KIRC (*p* = 9.9 × 10^−13^), and NT (*p* = 0.04), and a non-significant positive tendency for KIRP (*p* = 0.16).

**Figure 3:**
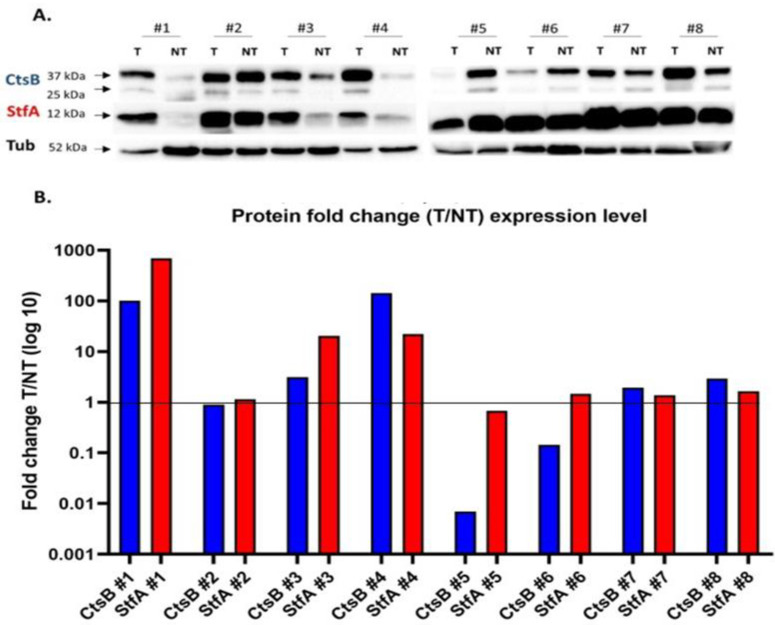
CtsB and StfA protein expression in renal cell carcinoma and adjacent healthy tissues. (**A**) CtsB and StfA protein expression was analyzed in the tissues by western blot. (**B**) Summary of the semiquantitative analysis of the band intensity normalized against tubulin (Tub). The figure represents the ratio between cancer tissue (T) and not tumor (NT) of CtsB or StfA in 8 pairs of tissues randomly selected in our sample collection.

To evaluate if the CtsB and StfA correlation expression was maintained at the protein level, we analyzed the expression of these proteins in eight randomly selected paired T and NT tissues (Figure 3). Total CtsB expression showed a solid increase in three samples out of eight and a mild increase in the other two samples. Similar results were obtained for the analysis of the only immature and mature forms of this protein (Supplementary Figure S3) and StfA. More importantly, total CtsB and StfA protein expression showed a positive correlation in seven samples out of eight. In one tumor sample, a robust CtsB expression decrease was not followed by a decrease in StfA expression. In the other samples, both the protein expressions in the tumor samples decreased, increased, or did not change compared to their expression in the adjacent non-cancerous tissue.

### 3.3. Effects on CtsB and StfA Expression and Cell Proliferation following Alternative Overexpression and Silencing In Vitro

A correlation between CtsB/StfA expression was confirmed in vitro using 769-P and A498 human RCC cells. We alternatively induced CtsB and StfA overexpression via cloned gene sequence construct transfections (pl*CTSB* and pl*STFA*, respectively). After cell treatment with the plasmids, we investigated CtsB and StfA protein expression compared to control cells treated with empty plasmids (CTRL) to evaluate if increased CtsB protein was followed by an increased StfA protein expression and vice versa.

**Figure 4:**
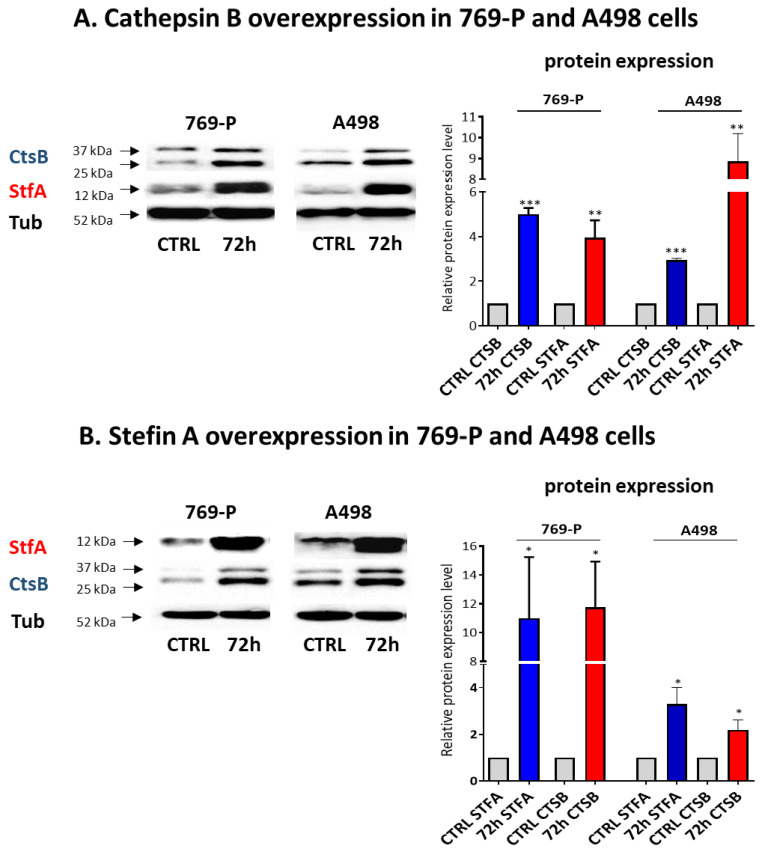
Overexpression of CtsB in RCC-derived cells induced StfA expression and vice versa. CtsB and StfA protein expression was determined by Western blotting in 769-P and A498 after transfection with (**A**) pl*CTSB* and (**B**) pl*STFA*. * = *p* < 0.05, ** = *p* < 0.01, *** = *p* < 0.001.

As hypothesized, the ectopic expression of CtsB and STFA induced StfA and CtsB expression, respectively, in both cell lines (Figure 4A,B).

**Figure 5:**
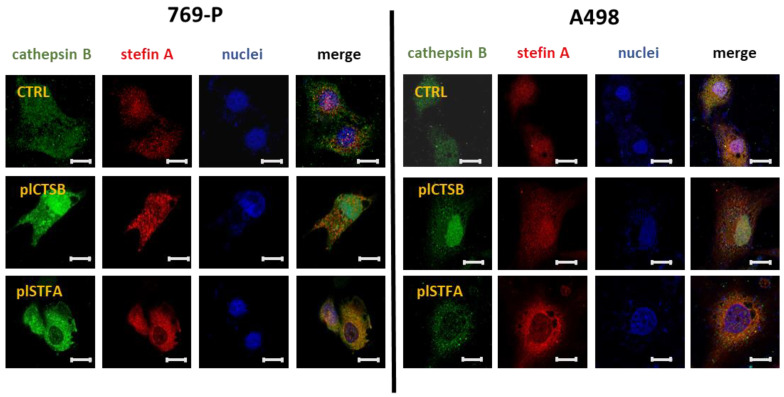
Effect of pl*CTSB* and pl*STFA* on StfA and CtsB expression, respectively, in RCC cell lines. After plasmid transfection, CtsB and StfA overexpression was evaluated by immunofluorescence staining. The cell lines were transfected for 72 h, fixed, and stained for CtsB (green) and StfA (red). DAPI staining was used to label the nucleus (blue). In both the cell lines, the overexpression of CtsB induced an increase in the cellular content of Stfa and vice versa. Representative images from three independent experiments are shown. Scale bar: 10 µm.

These data were confirmed by immunofluorescence labeling performed 72 h after transfection. Both CtsB and StfA were abundantly expressed in 769-P and A498 cells after transfection with pl*CTSB* and pl*STFA* (Figure 5). Interestingly while StfA increased within the cytoplasm of the cells, CtsB also appeared to localize in the nuclear area.

**Figure 6:**
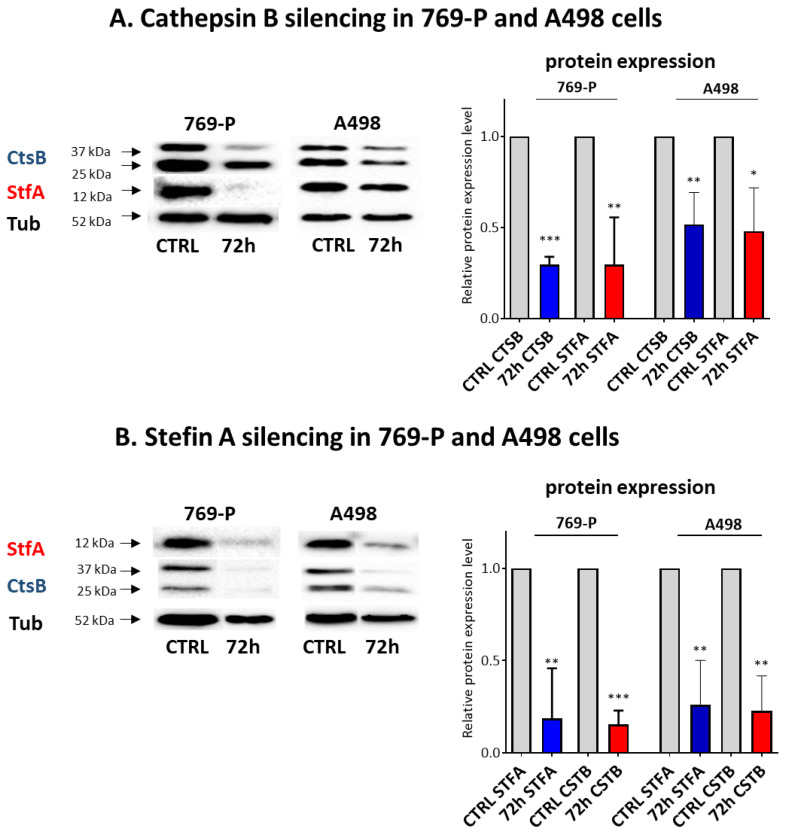
CtsB in RCC-derived cells reduced StfA expression and vice versa. CtsB and StfA protein expression was determined by Western blotting in 769-P and A498 after transfection with (**A**) sh*CTSB* and (**B**) sh*STFA* constructs compared to control cells treated with empty shRNA. The data were normalized against tubulin used as a housekeeping protein. All values were the average of at least three biological replicates, and the data represent the mean ± SD. The U Mann–Whitney test analyzed significance; * *p* < 0.05; ** *p* < 0.01, *** *p* < 0.001 relative to control.

To confirm the CtsB and StfA expression correlation, we investigated the reciprocal effect of CtsB and StfA silencing in RCC cells. We knocked down the expression of CtsB (Figure 6A) and StfA (Figure 6B) in 769-P and A498 cells using short hairpin RNA vectors (i) sh*CTSB* and (ii) sh*STFA*. Western blotting analysis showed that CtsB protein expression was strongly reduced after sh*CTSB* treatment compared to control cells transfected with empty shRNA. This observation was accompanied by a StfA protein reduction in the same samples. Similarly, StfA depletion resulted in CtsB protein downregulation in the same cell lines.

**Figure 7:**
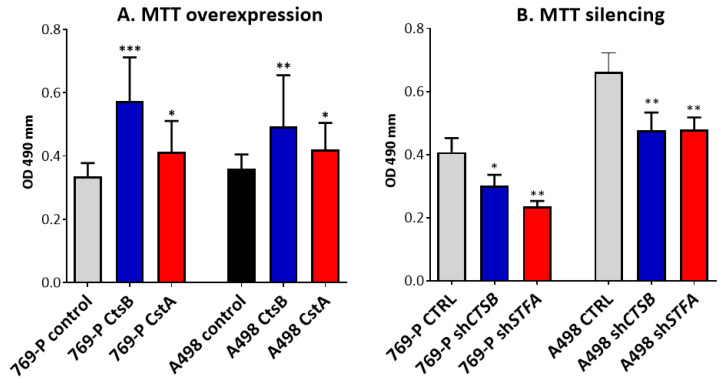
Effect of CtsB and StfA overexpression and silencing on the proliferation of RCC-derived cells. Cell proliferation following (**A**) CtsB/StfA overexpression and (**B**) CtsB/StfA silencing were analyzed through MTT assay. Proliferation was measured at 72 h after transfections of 769-P and A498 cells. Cells seeded in 96-well plates were treated with (3-(4,5-dimethylthiazol-2-yl)-2,5-diphenyltetrazolium bromide) (MTT) for 6 h at 37 °C. MTT was soluble and measured at 450 nm to determine the number of viable cells. Results were analyzed by the U Mann–Whitney test and presented as the mean ± SEM of at least three independent experiments; * *p* < 0.05, ** *p* < 0.01, and *** *p* < 0.001.

CtsB and StfA overexpression and silencing effects were evaluated on 769-P and A498 cell proliferation in comparison with control cells transfected with empty vectors (Figure 7A,B). As a result, we observed a significant growth increase in both cell lines after CtsB and StfA (769-P: pl*CTSB*, *p* = 0.001 and pl*STFA*, *p* = 0.026; A498: pl*CTSB*, *p* = 0.006, pl*STFA*, *p* = 0.043) overexpression.

Consistently, CtsB and StfA silencing negatively affected cell proliferation (769-P: sh*CTSB*, *p* = 0.016; sh*STFA*, *p* = 0.003; A498: sh*CTSB*, *p* = 0.009; sh*STFA, p* = 0.0022).

A further investigation was performed to evaluate the expression of Snail1 after CtsB and StfA overexpression. Snail1 expression correlates with the enhanced invasion and mobility of tumor cells and it is also one of the epithelial to mesenchymal transition biomarkers [39]. However, Snail1 has also been shown to reduce the apoptosis rate in cancer cells [39,40]. Snail1 slightly increased both in 769-P and A498 cells after StfA overexpression, but this trend was not confirmed after CtsB overexpression (Supplementary Figure S4).

## 4. Discussion

RCC is a heterogeneous group of epithelial malignancies characterized by a specific biology, clinical manifestations, and prognostic outcomes [41]. Understanding the molecular pathways involved in RCC pathogenesis can support new biomarker discovery and targeted treatment development [42]. One factor contributing to the malignancy of solid tumors is the low intra- and extracellular pH [43]. The acid-mediated cancer cell invasion can favor Cts-mediated basement membrane degradation, as described in melanoma [44,45], ovarian [46], breast cancer [47], and as well as RCC [48].

Cts expression is unregulated in many tumors and is potentially involved in the early premalignant process, cancer progression, angiogenesis, metastasis, and drug resistance [9,49]. Cts activity can be regulated by natural inhibitors [18] that can interact with their active site reversibly or irreversibly [50]. It has been reported that alterations between the ratio of cathepsins and their inhibitors may result in metastasis and invasion in the prostate [51], breast [52], brain [53], colorectal [54], and head and neck [55] cancers.

Typically, increased CtsB activity is related to its increased expression and decreased expression of stefins [56]. On the other hand, StfA has differentially been identified as a suppressor but also as an oncoprotein in many human tumors, and its low expression has correlated with a better outcome in breast [34], liver [35], and brain [36] cancers. It is believed that each cathepsin and stefin member has relatively different functions in normal and diseased conditions. Thus, an investigation of the cathepsins–stefins correlation and regulation need to be explored for a cancer tissue-specific context.

Consistent with the findings described here, many studies have demonstrated that a high expression level of CtsB and StfA was associated with cancer, higher tumor grade, and poor prognosis in patients [34,35,36,53,55,57,58,59]; however, the CtsB and StfA relation in RCC was not checked. The significance of CtsB in RCC was explored by Bhatt R.S. et al. [26], who showed that as a result of CtsB expression inhibition, an RCC growth inhibition in vitro and in vivo was registered. In addition, the quantitative proteome profiling on xenograft tumors indicated that CtsB is clinically involved in RCC sunitinib resistance [26]. However, its expression concerning its natural inhibitor StfA in RCC was never investigated.

CtsB and StfA mRNA and protein distribution were evaluated by in situ hybridization [60] and immunostaining [51,61], respectively, in prostate cancer samples. Compared to normal prostate and benign prostatic hyperplasia, a positive CtsB/StfA ratio was showed to be a potential marker for more aggressive variants of prostate cancer according to the Gleason histologic score [60,61] and for the incidence of pelvic lymph node metastases [51]. Overall, these data show that an increase in the CtsB/STFA ratio (higher CtsB expression accompanied by StfA impoverishment) can cause a more aggressive tumor phenotype.

The current study aimed to determine *CTSB* and *STFA* mRNA expression between RCC cancer lesions and surrounding healthy renal tissue. The results demonstrated that *CTSB* and *STFA* mRNA expression significantly increased in tumoral samples. In addition, *CTSB* expression correlated with *STFA* in both T and NT tissues (Spearman’s rank correlation; R *CTSB* = 0.689, R *STFA* = 0.570, *p* < 0.05). It was also determined that *CTSB* expression increased significantly in KIRC, and KICH compared to AML, which presents a lower malignant behavior. Additionally, the analyzed *CTSB*/*STFA* ratio showed lower values in the tumor samples due to the higher expression of the inhibitor in non-cancerous tissues. Therefore, our study did not confirm the general rule of an increased protease/inhibitor ratio in the tumor.

Next, the higher expression of *CTSB* and *STFA* mRNAs was associated with the cases of cancer affecting both kidneys compared to patients with single kidney involvement (right or left kidney).

Our analysis showed that *CTSB* expression in the tumor tissues of patients without metastasis (M0) positively correlated with *STFA* expression (R = 0.764, *p* < 0.05). In patients with metastasis (M1), the *CTSB* in T tissues showed a strong negative correlation with *STFA* in adjacent non-cancerous tissues (R = −0.886, *p* < 0.05). This negative relation of CtsB in metastasizing RCC and StfA in paired non-cancer margins may suggest that tumor progression leads to the active changes in CtsB expression with decreased inhibition. We suppose that a lower inhibitor activity in the surrounding cancer accompanied by enhancing the presence of CtsB in the cancer core can facilitate the cancer cell spreading [53].

Further, a significant CtsB enhancement was registered in normal kidney tissues of elders, and this phenomenon could be connected with the age-related role of CtsB that was previously described in human serum [62] and a rat’s brain [63] and liver [64].

Significantly, in most analyzed tissues, regardless of the clinical factors, CtsB expression positively correlated with StfA expression level in ~85% of analyzed tissue samples (tumors vs. normal tissues) at the transcriptomic and proteomic levels. The correlation between CtsB and StfA expression in RCC and surrounding normal kidney tissues was confirmed by the information available from the GEPIA database. These observations suggest a strong dependency between these two molecules. To confirm this observation, we overexpressed and silenced CtsB and StfA in RCC-derived cell lines 769-P and A498. In both cell lines, the overexpression or silencing of CtsB enhanced or reduced StfA expression and vice versa. However, this phenomenon should be further investigated to define the molecular mechanism at the base of this regulation.

The immunofluorescence staining showed that ectopic CtsB was abundantly expressed in nuclear and cytoplasmic fractions. We believe our data reflect the natural expression of CtsB both in the cytoplasmic and nuclear compartments, whereas StfA presence was detected only in the cytoplasm. CtsB was detected in varied organelles, including the mitochondria [65], nucleus [66], and cytosol [67] regulating cell death [67,68] and cellular division [66]. On the other hand, StfA was detected outside the lysosomes in the peripheral cytoplasm and the plasma membrane region [63].

The study presented by Baici A et al., 2006 [69] using GFP-tagged CtsB showed that the proper regulation and distribution of CtsB is vital for cellular functioning, and any aberrant expression of CtsB can disrupt the cell homeostasis and result in a malignant phenotype.

By increasing CtsB or StfA expression in 769-P and A498 cells, we registered an increase in RCC cell proliferation, with the more substantial change observed with CtsB overexpression. In our previous work [70], we demonstrated that peptidic inhibitors of cysteine cathepsins could increase the protein expression of Snail1. This transcription factor was shown to inhibit cell apoptosis [39,40] and we hypothesized its involvement in the increased proliferation observed after StfA overexpression and indirectly after CtsB overexpression, since this treatment also increased the StfA level. Accordingly, we observed a slight increase in Snail1 after StfA overexpression in both cell lines, but these data were not confirmed after CtsB overexpression, indicating that more research is needed to understand the working mechanism of this phenomenon. A higher CtsB expression in mammary cancer cells increased cell division [71,72,73], while its silencing in glioblastoma inhibited cell proliferation, reducing the levels of pERK and pFAK [74,75]. No data have pointed out the regulatory role of StfA in tumor cells; however, StfA was detected as a critical molecule in a common inflammatory disease of the skin (psoriasis Vulgaris), characterized by hyperproliferation of skin cells [76]. In addition, other stefins, CtsB and CtsC, actively regulate cancer cell proliferation [77,78]. In contrast, the silencing of CtsB and StfA significantly reduced the growth of RCC-derived cells, highlighting the importance of this balance in cancer cell biology.

## 5. Conclusions

Taken together, the present study is the first to investigate both CtsB and StfA expression in RCC human samples. Increased expression of CtsB and StfA in RCC vs. non-tumoral and benign tissue samples might indicate a role in the development and progression of this disease. Furthermore, the in vitro experiments using RCC-derived cells confirmed the positive expressive correlation between CtsB and StfA and their effect on the proliferation of RCC cells. Thus, our observations have emphasized the role of CtsB and StfA in RCC development and future investigations will be worthwhile to further clarify their prognostic, diagnostic, and therapeutic potentials. In particular, a thorough analysis of the protein expression of this protease and its activity could definitely reveal its role in the progression of this disease.

## Figures and Tables

**Table 1:** Spearman’s rank correlation of *CTSB* and *STFA* mRNA expression with clinical and pathological features of human renal cell carcinomas.

Terms of Spearman’s Rank Correlation	Location	Correlation	R-Value	*p*-Value
*CTSB* vs. *STFA* expression	T	Positive	R = 0.689	*p* < 0.05
*CTSB* vs. *STFA* expression	NT	Positive	R = 0.570	*p* < 0.05
*CTSB* vs. *STFA* expression	T in M0 patients	Positive	R = 0.764	*p* < 0.05
*CTSB* (T) vs. *STFA* (NT) expression	T, NT in M1 patients	Negative	R = −0.886	*p* < 0.05

## Data Availability

The data presented in this study are available on request from the corresponding author. The data is not publicly available due to privacy restrictions.

## Supplementary Materials

The following supporting information can be downloaded at: https://www.mdpi.com/article/10.3390/cells11091455/s1, Figure S1: A. Native form of cathepsin B (CtsB) includes 17 amino acids of a signal peptide, 62 amino acids of the propeptide, and 260 amino acids of mature form. The signal peptide mediates translocation of CtsB into the rough endoplasmic reticulum, where it is cleaved and pro-cathepsin is formed. The pre-part is translocated into Golgi-apparatus, and then the residues: 38th-Asn and the 111th-Asn are glycosylated by high-mannose-type sugar. The phosphorylated protein binds to a mannose-6-phosphate receptor in the trans-Golgi network and is transported to lysosomes. In the low pH of lysosomes, pro-CtsB undergoes autocatalytic activation, leading to active cathepsin B formation. Alternatively, CtsB can be activated by an aspartic protease-CtsD. A proteolytic cleavage between residues 47 and 50 generates the double chain of heavy and light chains. B. CtsB in complex with stefin A; PDB 3K9M; Figure S2: Cancer-specific survival (in years) of renal cell carcinoma patients in association with cathepsin B (CTSB) and stefin A (STFA) expression. Kaplan-Meier survival curve showing the relation between low and high A. CTSB and B. STFA expression levels in chromophobe (KICH), papillary (KIRP) and clear cell (KIRC) renal cell carcinomas. From TCGA (The Cancer Genome Atlas) database (https://tcga-data.nci.nih.gov/tcga/ accessed on 1 January 2022), the survival curves showed that higher expression of CTSB had a significantly poor survival in 405 KICH patients (*p* = 0.0015), 119 KIRP (*p* = 0.021) and 422 KIRC (*p* = 0.29). Next, up-regulation of STFA showed worse survival trend in 49 KICH (*p* = 0.18) and significantly worse survival time 57 KIRP (*p* = 0.039) and 111 KIRC (*p* = 0.0006); Figure S3: Summary of semiquantitative analysis of the band intensity normalized against tubulin (Tub). The figure represents the ratio of immature and mature CtsB collected in tumor (T) and not tumor (NT) samples obtained from the same patient, in eight randomly selected pairs of tissues; Figure S4: Expression of Snail1 after overexpression of CtsB and StfA in 769-P and A498 cell lines. Cells were treated with empty plasmid (CTRL) and plCTSB, plSTFA constructs. Snail1 protein expression level was determined 72 h after transfection by Western blotting; tubulin served as the housekeeping protein; Table S1: Patient clinical and pathological features and CTSB and STFA expression. The relative quantification value (RQ) was calculated as the relative change of the transcript expression normalized against the internal control represented by a mixture of RNAs extracted from control samples (*n* = 43).
