# Supplementary material for: In Silico, In Vitro, and Clinical Investigations of Cathepsin B and Stefin A mRNA Expression and a Correlation Analysis in Kidney Cancer
Source: Cells. 2022 Apr 25;11(9):1455. doi: 10.3390/cells11091455 (PMC9101197; doi:10.3390/cells11091455)
Supplement: Supplementary file 1 [file cells-11-01455-s001.zip › cells-1635583-supplementary.pdf]

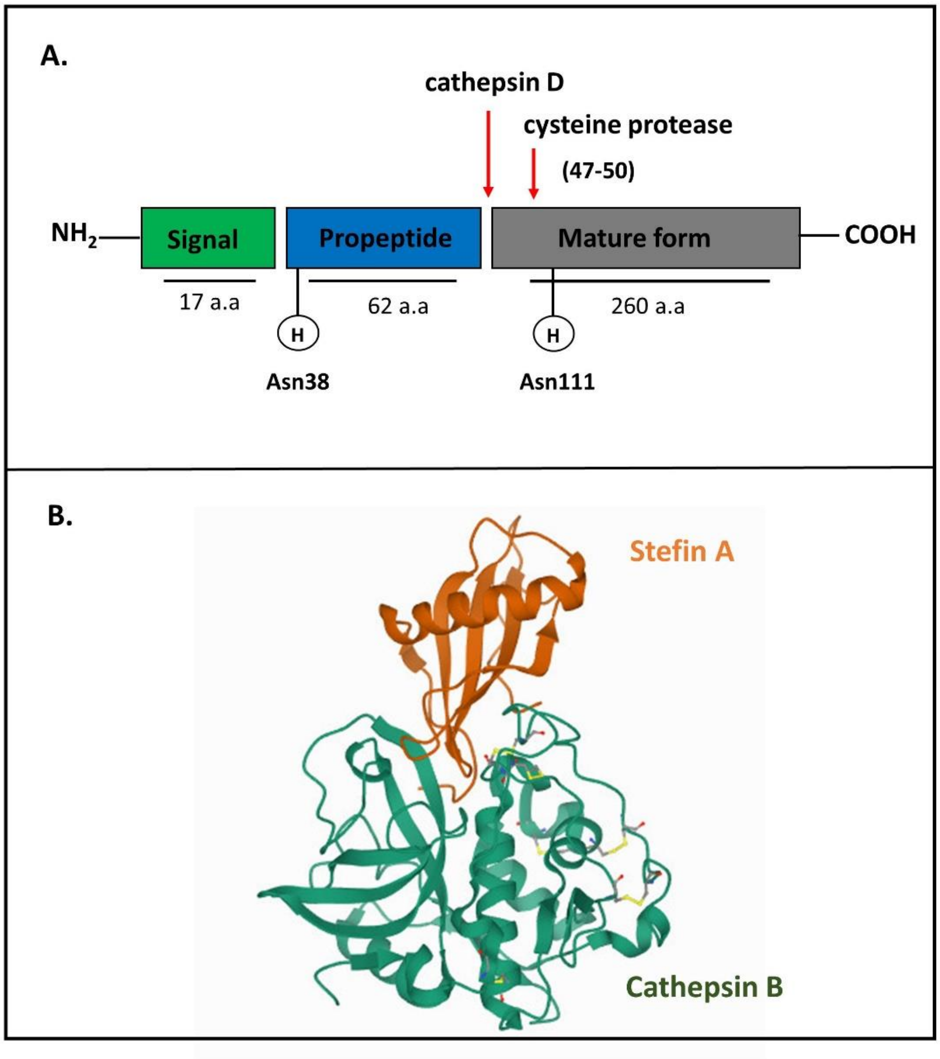

Figure S1. (**A**). Native form of cathepsin (**B**) (CtsB) includes 17 amino acids of a signal peptide, 62 amino acids of the propeptide, and 260 amino acids of mature form. The signal peptide mediates translocation of CtsB into the rough endoplasmic reticulum, where it is cleaved and pro-cathepsin is formed. The pre-part is translocated into Golgi-apparatus, and then the residues: 38th-Asn and the 111th-Asn are glycosylated by high-mannose-type sugar. The phosphorylated protein binds to a mannose-6-phosphate receptor in the trans-Golgi network and is transported to lysosomes. In the low pH of lysosomes, pro-CtsB undergoes autocatalytic activation, leading to active cathepsin B formation. Alternatively, CtsB can be activated by an aspartic protease - CtsD. (**A**) proteolytic cleavage between residues 47 and 50 generates the double chain of heavy and light chains. (**B**) CtsB in complex with stefin A; PDB 3K9M.

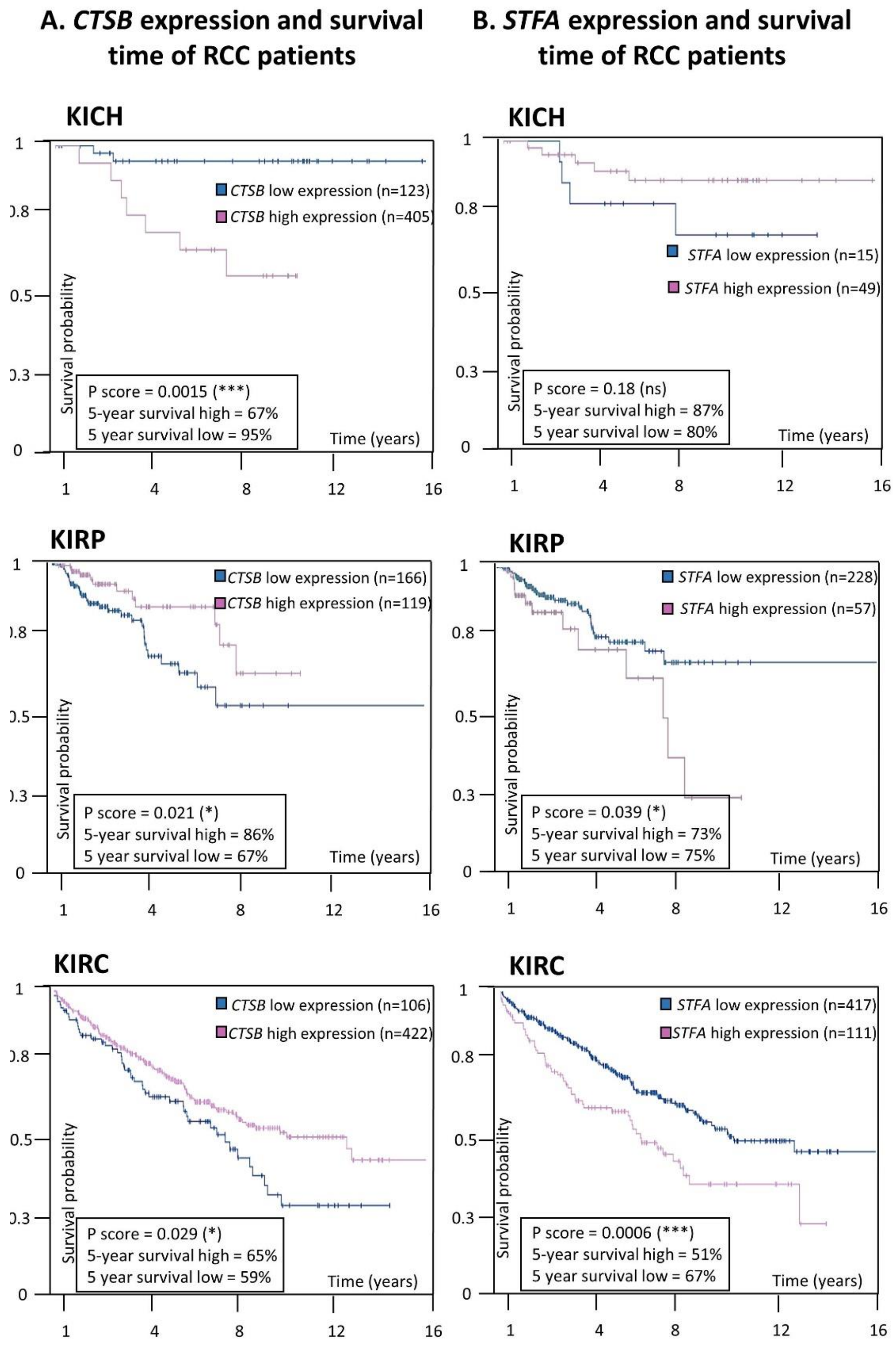

Figure S2. Cancer-specific survival (in years) of renal cell carcinoma patients in association with cathepsin (**B**) (CTSB) and stefin (**A**) (STFA) expression. Kaplan-Meier survival curve showing the relation between low and high A. CTSB and B. STFA expression levels in chromophobe (KICH), papillary (KIRP) and clear

cell (KIRC) renal cell carcinomas. From TCGA (The Cancer Genome Atlas) database (https://tcga-data.nci.nih.gov/tcga/), the survival curves showed that higher expression of CTSB had a significantly poor survival in 405 KICH patients (p = 0.0015), 119 KIRP (p = 0.021) and 422 KIRC (p = 0.29). Next, up-regulation of STFA showed worse survival trend in 49 KICH (p = 0.18) and significantly worse survival time 57 KIRP (p = 0.039) and 111 KIRC (p = 0.0006).

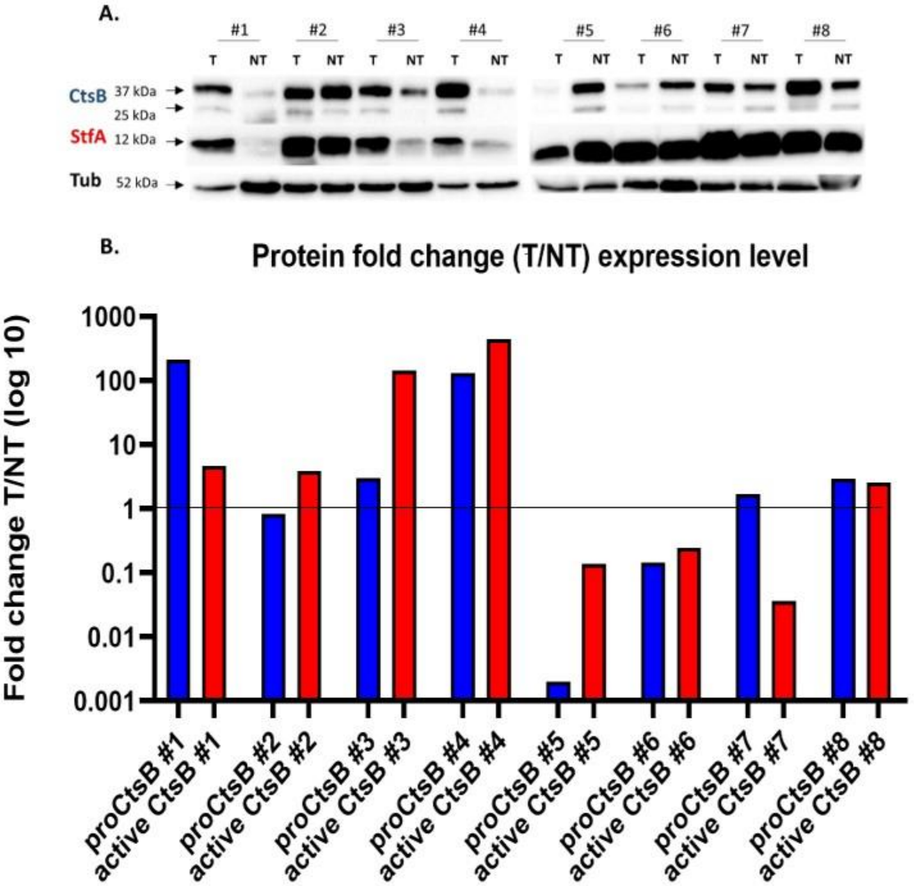

Figure S3. Summary of semiquantitative analysis of the band intensity normalized against tubulin (Tub). (**A**,**B**) The figure represents the ratio of immature and mature CtsB collected in tumor (T) and not tumor (NT) samples obtained from the same patient, in eight randomly selected pairs of tissues.

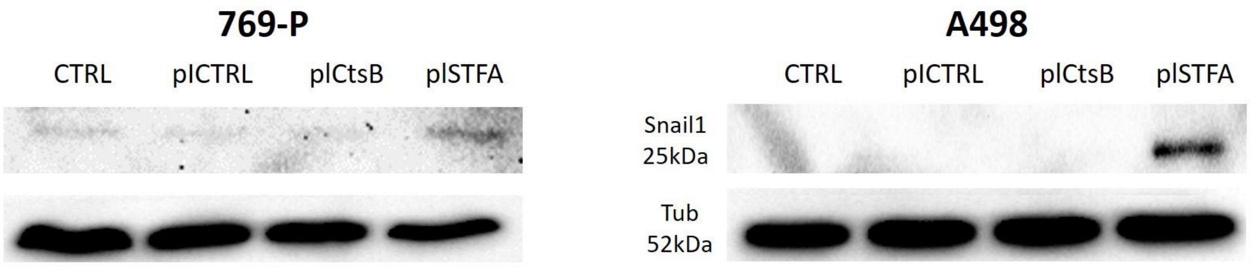

Figure S4. Expression of Snail1 after overexpression of CtsB and StfA in 769-P and A498 cell lines. Cells were treated with empty plasmid (CTRL) and plCTSB, plSTFA constructs. Snail1 protein expression level was determined 72h after transfection by Western blotting; tubulin served as the housekeeping protein.

Table S1. Patient clinical and pathological features and *CTSB* and *STFA* expression. The relative quantification value (RQ) was calculated as the relative change of the transcript expression normalized against the internal control represented by a mixture of RNAs extracted from control samples (n = 43).

**Clinical and pathological features**			***CTSB* T**		***CTSB* NT**		***STFA* T**		***STFA* NT**	
		N	Mean	±SD	Mean	±SD	Mean	±SD	Mean	±SD
**Entire group**		43	12.80	20.41	4.50	5.76	15.53	29.79	7.81	18.29
**Gender**	**Men**	22	14.18	23.20	4.89	5.38	14.20	31.01	11.36	24.37
	**Woman**	21	11.36	17.48	4.09	6.24	16.91	29.15	4.09	7.11
**Age group**	**≤ 60 yrs**	22	12.62	18.39	3.47	5.50	18.28	32.21	9.96	24.72
	**> 60 yrs**	21	12.99	22.80	5.57	5.97	12.64	27.52	5.56	7.10
**Histopathological type**	**KIRC**	33	13.84	21.31	3.91	3.92	16.54	32.44	6.54	12.98
	**KIRP**	3	27.27	26.73	9.64	14.10	12.66	13.77	34.94	55.47
	**KICH**	4	2.72	1.49	7.88	10.59	17.55	30.21	1.93	2.29
	**AML**	3	0.36	0.07	1.30	0.38	4.55	4.01	2.48	1.67
***pTNM**	**pT1-T2**	26	9.73	15.99	5.33	7.01	12.95	25.78	7.98	19.30

	**Tumor size**	**pT3 –pT4**	14	12.84	17.83	3.13	2.26	20.60	38.61	8.30	18,17
	**Lymph node invasion**	**pN0**	38	13.98	21.38	4.80	6.05	16.24	31.54	8.50	19,37
		**pN1**	2	8.99	6.81	3.62	2.00	18.33	4.90	2.72	1,80
	**Metastasis**	**pM0**	34	14.06	21.10	4.80	6.32	17.44	32.62	8.51	20,21
		**pM1**	6	11.86	21.38	4.37	2.88	10.16	17.15	6.50	9.47
**Presence in one/both kidneys**		**One lobe**	39	10.75	16.83	4.71	6.01	13.15	25.72	8.47	19.10
		**Both lobes**	4	32.76	40.75	2.44	1.11	38.68	57.07	1.37	0.93
****Grade**		**G1**	18	12.37	24.92	4.61	4.80	13.33	33.50	4.12	6.55
		**G2**	17	17.65	18.87	5.28	7.69	21.70	32.15	13.37	27.70
		**G3-G4**	5	5.34	4.98	3.36	1.47	9.04	8.86	5.42	6.16

KIRC - clear renal cell carcinoma, KIRP - papillary renal cell carcinoma, KICH – chromophobe renal cell carcinoma, AML - renal angiomyolipoma (benign renal neoplasm).
*pTNM- International System of Clinico-Morphological Classification of Tumours (TNM - Tumour Node Metastasis), pT1: tumor confined to kidney > 4 cm but < 7 cm, pT2: limited to kidney > 7 cm, pT3: tumor extension into major veins or perinephric tissues, but not into ipsilateral adrenal gland or beyond Gerota's fascia, pT4: involves ipsilateral adrenal gland or invades beyond Gerota's fascia. The number of patients included in this classification is 40. Three patients were not considered because they were affected by AML subtype, not included in tumor grade staging classification
**Grade: G1: well-differentiated (low grade), G2: moderately differentiated (intermediate grade), G3: poorly differentiated (high grade), G4: undifferentiated (high grade).
